# Le côté obscur de l’éclaircissement de la peau

**DOI:** 10.48327/mtsi.v2i4.2022.264

**Published:** 2022-10-27

**Authors:** Dorian CELLARIER, Alisson ADET, Mathilde BARRÉ

**Affiliations:** 1 154^e^ antenne médicale (AM) d'Aubagne, 10^e^ Centre médical des Armées (CMA), Marseille, France; 2 Service médical de la Force d'action navale de Toulon, France; 3 Service de dermatologie, Hôpital Nord, Marseille, France

En République démocratique du Congo, un patient de 44 ans, sans antécédent et vivant à Kinshasa, consultait pour des lésions cutanées des coudes (Fig. 1 et Fig. 2) douloureuses avec une sensation de brûlure. Il rapportait l'utilisation d'une lotion dépigmentante achetée sur son marché local, annoncée comme « correcteur de tâche ». Après une application biquotidienne de cette préparation sur une hyperpigmentation ancienne des coudes, il présentait une dermite de contact érosive d'apparition progressive en regard des zones d'application.

**Figure 1:**
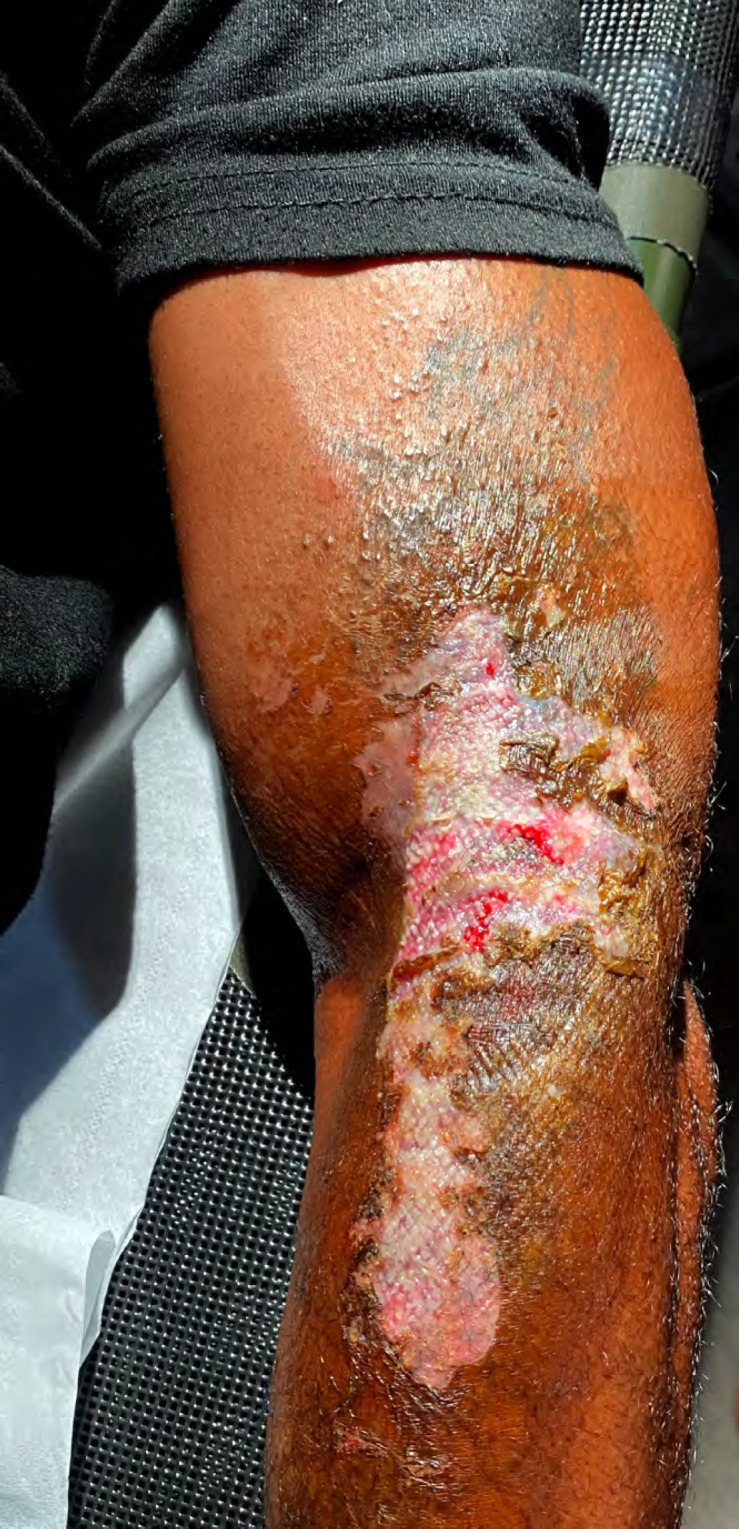
Lésion bras gauche à J1 Left arm lesion at D1

**Figure 2:**
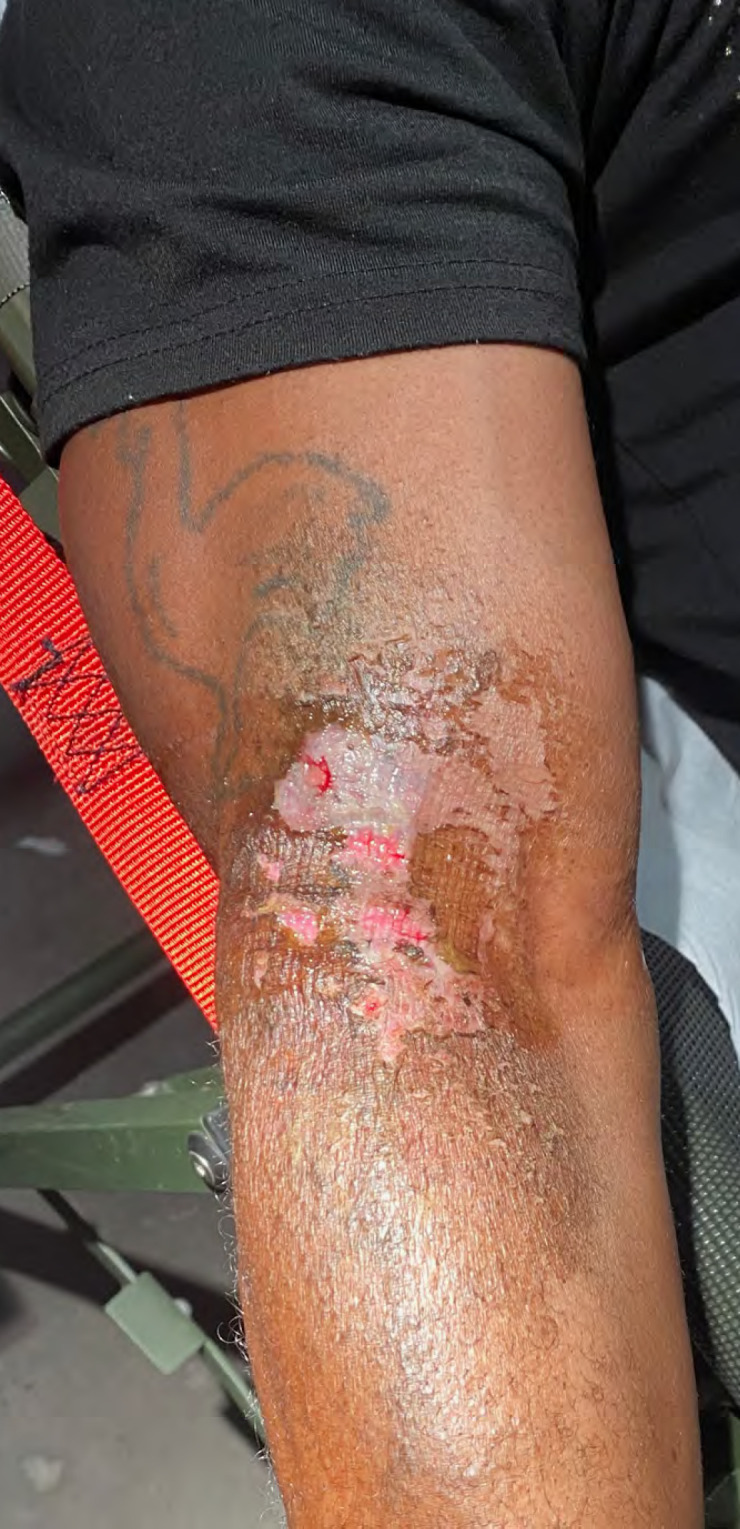
Lésion bras droit à J1 Right arm lesion at D1

Le patient a bénéficié d'un protocole de soins quotidiens qui consistait en une désinfection en 4 temps, suivi d'une application de sulfadiazine argentique (Flammazine^®^) et de tulle gras (Jelonet^®^). Après 15 jours de soins, la cicatrisation était satisfaisante avec ré-épidermisation progressive des zones atteintes, mais persistance de zones d'atrophie cutanée associées à une dyschromie post-inflammatoire résiduelle (Fig. 3 et 4).

**Figure 3:**
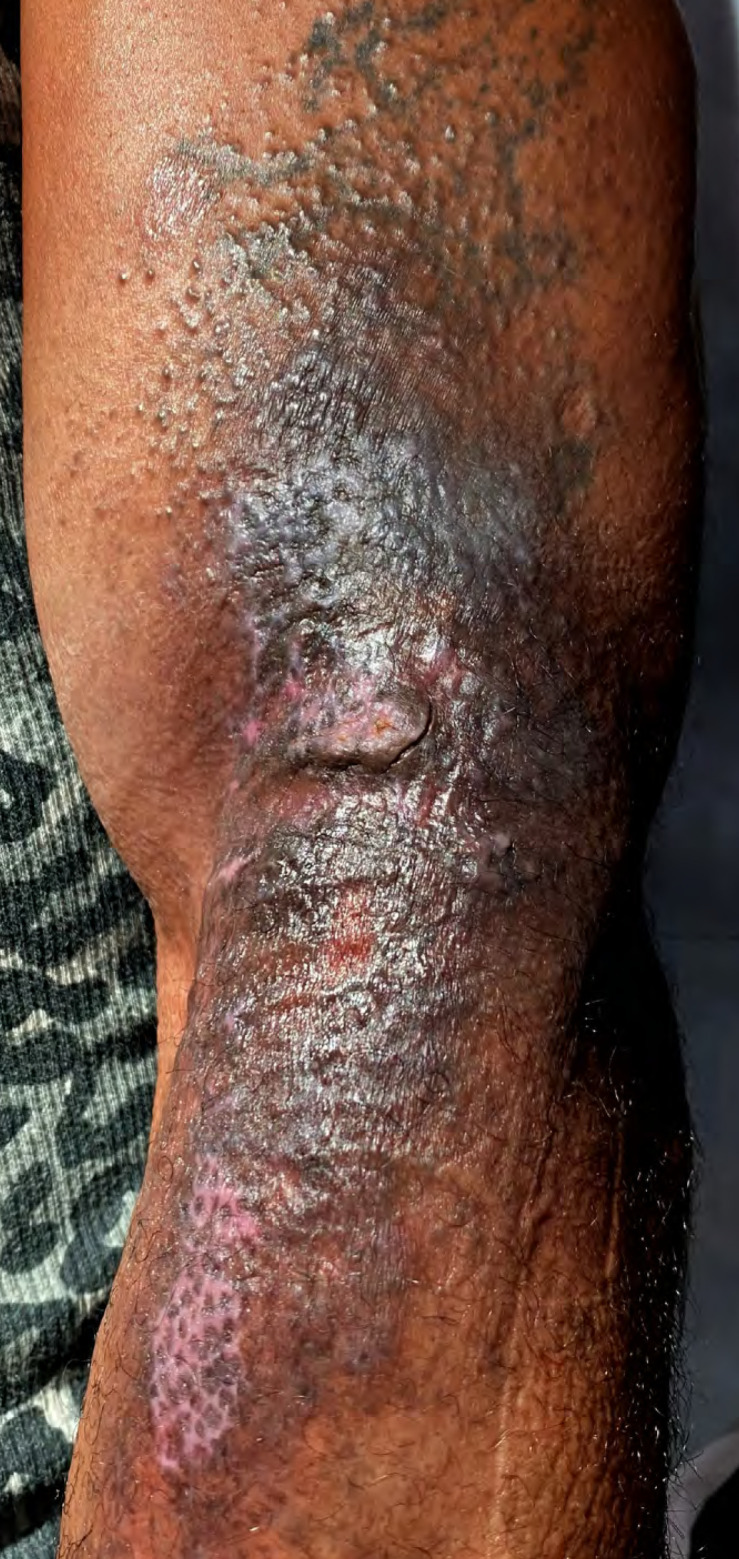
Lésion bras gauche à J15 Left arm lesion at D1

**Figure 4:**
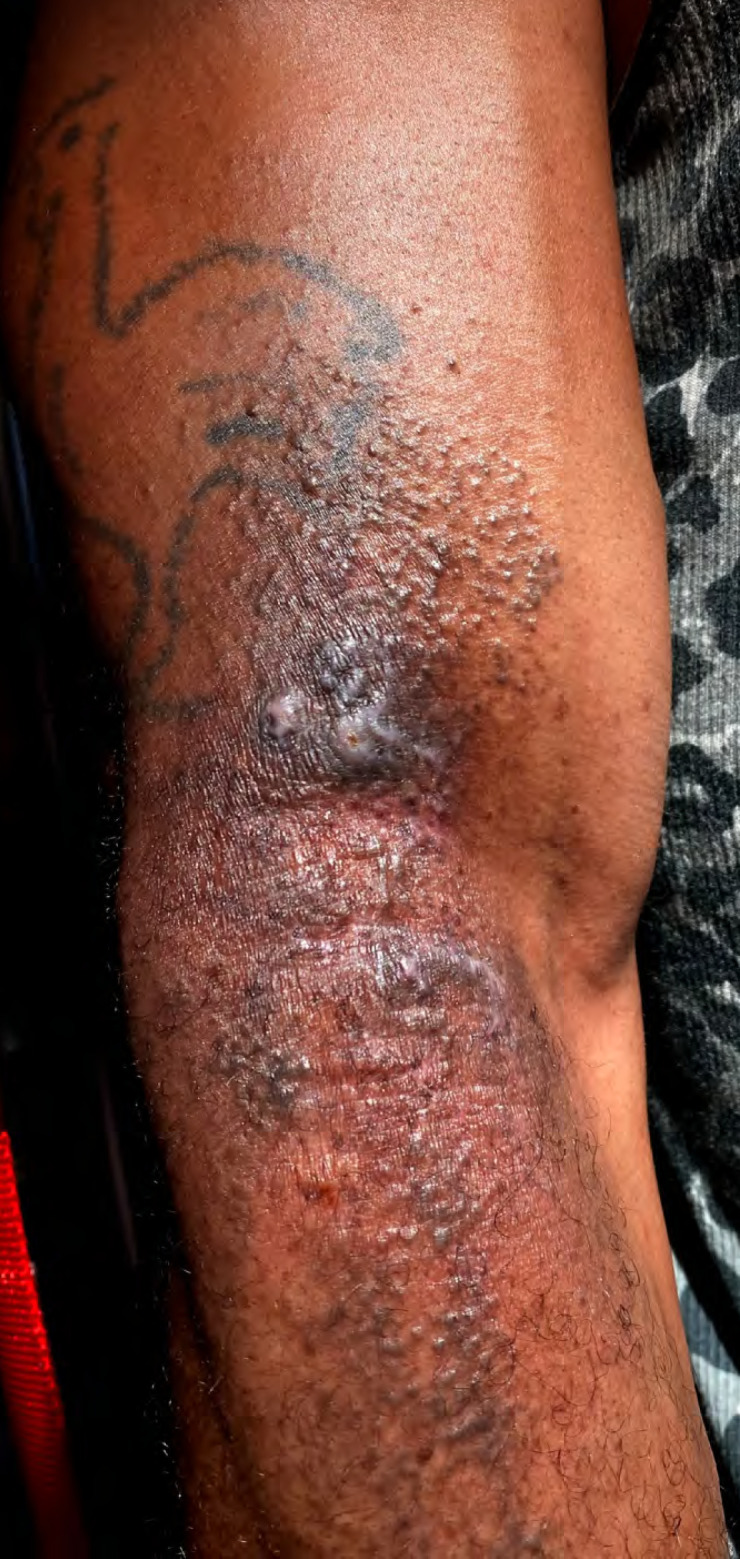
Lésion bras droit à J15 Right arm lesion at D15

Parmi les ingrédients mentionnés dans le produit utilisé (Fig. 5), on retrouvait notamment de l'hydroquinone 2% et du propionate de clobétasol, un dermocorticoïde d'activité très forte dont l'utilisation est limitée aux dermatoses inflammatoires lichénifiées et aux dermatoses bulleuses auto-immunes. La lotion ne semblait pas contenir de mercure, mais la composition exacte n'a pas pu être définie précisément dans un laboratoire. Avec une provenance et une composition potentiellement douteuse du cosmétique, le risque de contrefaçon ou de malfaçon du produit en lui-même (volontaire ou involontaire) était majeur.

**Figure 5:**
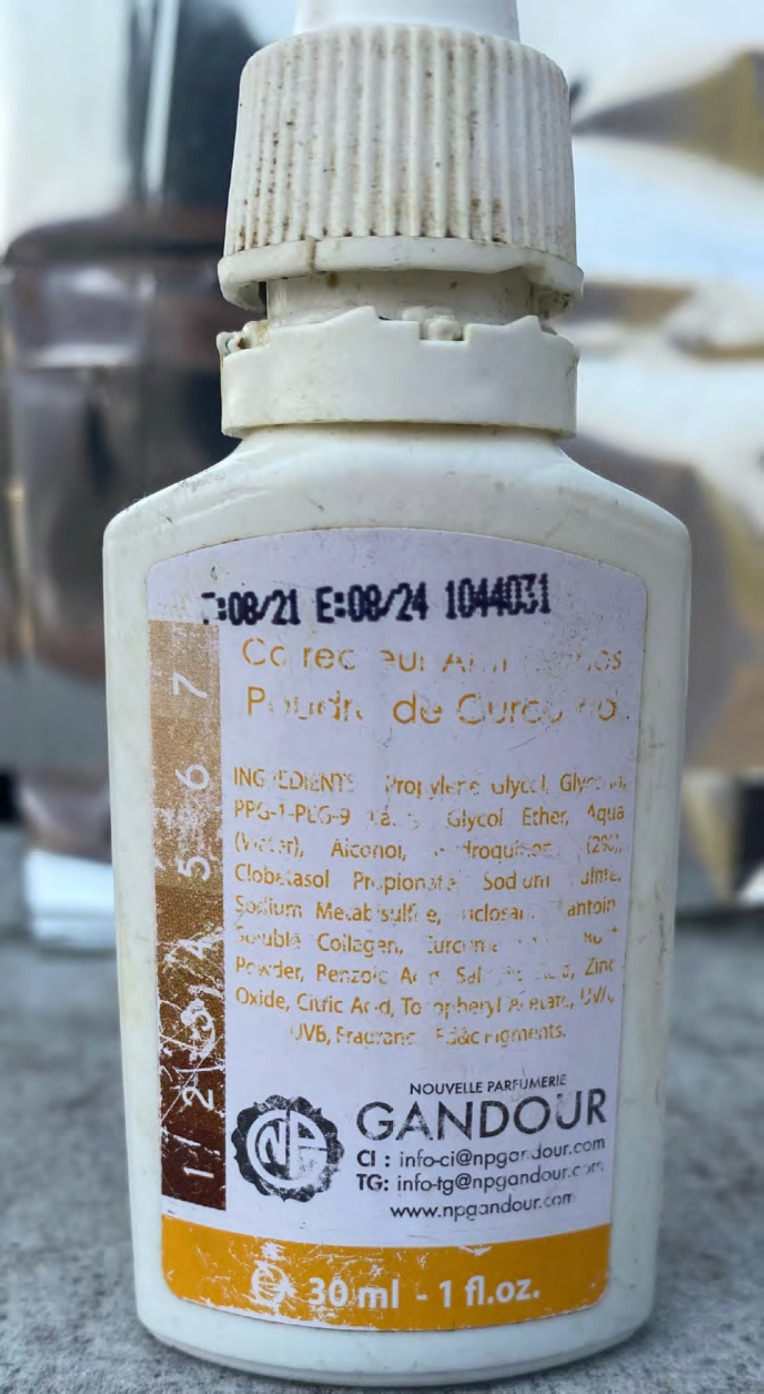
Lotion antitache achetée sur un marché Anti-stain lotion bought on a market

Malgré les dangers à court et long termes démontrés des produits cosmétiques dépigmentants [2], le phénomène culturel et social du paraître « plus clair » n'est pas nouveau [1]. Toujours d'actualité dans de nombreux pays et notamment en Afrique subsaharienne, où la publicité pour ces produits reste présente, le phénomène de *skin bleaching,* via l'utilisation de substances telles que le mercure, l'hydroquinone ou encore les glucocorticoïdes, n'est pas sans risque pour la peau et la santé de ses utilisateurs.

Pour rappel l'hydroquinone, molécule dépigmentante interdite dans les cosmétiques depuis 2001 dans l'Union européenne, est encore parfois retrouvée dans un certain nombre de produits illicites et peut provoquer entre autres des eczémas de contact et des irritations lors d'applications répétées, ou encore une dyschromie irréversible. Enfin, une utilisation prolongée ou de trop forte concentration d'hydroquinone peut causer une hyperpigmentation paradoxale qu'on appelle ochronose exogène. L'utilisation de dermocorticoïdes est réservée au traitement des dermatoses inflammatoires telles que l'eczéma ou le psoriasis. L'utilisation de mercure dans la quasi-totalité des savons et cosmétiques est en revanche interdite par l'ONU depuis 2013. À noter que l'hydroquinone est encore de nos jours utilisée en association avec la trétinoïne et la dexaméthasone dans le traitement médical de l'hyperpigmentation, sous réserve d'une prescription médicale et d'une surveillance clinique régulière.

Les conditions d'accès précaires aux produits pharmaceutiques et cosmétiques de qualité normée et contrôlée s'ajoutent au risque d'obtention d'un dangereux cocktail « *skin bleaching* ».

La prévention et l’éducation des populations concernées restent donc un enjeu majeur concernant l'utilisation non médicale de substances dépigmentantes. Il est important que le personnel médical soit informé des risques à court et à long terme de ces molécules pharmaceutiques, afin de contribuer à l’éducation des populations et réduire le risque de mésusage sur l'ensemble du globe [3].

## Liens D'intérêts

Les auteurs ne déclarent aucun lien d'intérêt.

## Contribution Des Auteurs

Dorian CELLARIER: Prise en charge et rédaction

Alisson ADET: Correction

Mathilde BARRÉ: Prise en charge, correction et rédaction
